# Autophagy protects against palmitate-induced apoptosis in hepatocytes

**DOI:** 10.1186/2045-3701-4-28

**Published:** 2014-05-21

**Authors:** Ning Cai, Xue Zhao, Yingying Jing, Kai Sun, Shufan Jiao, Xiaojing Chen, Haozheng Yang, Yan Zhou, Lixin Wei

**Affiliations:** 1 Medical Sciences Research Center, Ren Ji hospital, School of Medicine, Shanghai Jiao Tong University, Shanghai, China; 2 Tumor Immunology and Gene Therapy Center, Eastern Hepatobiliary Surgery Hospital, The Second Military Medical University, Shanghai, China

**Keywords:** Autophagy, Palmitate, Hepatocytes, Apoptosis, Protector

## Abstract

**Background:**

Non-alcoholic fatty liver disease, one of the most common liver diseases, has obtained increasing attention. Palmitate (PA)-induced liver injury is considered a risk factor for the development of non-alcoholic fatty liver disease. Autophagy, a cellular degradative pathway, is an important self-defense mechanism in response to various stresses. In this study, we investigated whether autophagy plays a protective role in the progression of PA-induced hepatocytes injury.

**Results:**

Annexin V-FITC/PI staining by FCM analysis, TUNEL assay and the detection of PARP and cleaved caspase3 expression levels demonstrated that PA treatment prominently induced the apoptosis of hepatocytes. Meanwhile, treatment of PA strongly induced the formation of GFP-LC3 dots, the conversion from LC3I to LC3II, the decrease of p62 protein levels and the increase of autophagosomes. These results indicated that PA also induced autophagy activation. Autophagy inhibition through chloroquine pretreatment or Atg5shRNA infection led to the increase of cell apoptosis after PA treatment. Moreover, induction of autophagy by pretreatment with rapamycin resulted in distinct decrease of PA-induced apoptosis. Therefore, autophagy can prevent hepatocytes from PA-induced apoptosis. In the further study, we explored pathway of autophagy activation in PA-treated hepatocytes. We found that PA activated PKCα in hepatocytes, and had no influence on mammalian target of rapamycin and endoplasmic reticulum stress pathways.

**Conclusions:**

These results demonstrated that autophagy plays a protective role in PA-induced hepatocytes apoptosis. And PA might induce autophagy through activating PKCα pathway in hepatocytes.

## Introduction

Non-alcoholic fatty liver disease (NAFLD) is usually considered the accumulation of extra fat in hepatocytes that is not caused by alcohol [1]. In recent years, its incidence is rapidly rising and affects not only adults, but also children [2,3]. NAFLD refers to a spectrum of disease ranging from steatosis to inflammation in nonalcoholic steatohepatitis (NASH) with different degrees of fibrosis that can progress to cirrhosis [4-6]. Accumulating evidence suggests that it is implicated with the levels of plasma free fatty acids (FFAs), the primary source for triacylglycerols (TAGs) in hepatocytes [3,7-9]. Some studies demonstrated the condition that hepatocytes were exposed to elevated FFAs could promote steatosis and hepatic apoptosis via activation of Bim and PUMA [10,11]. Hepatocytes apoptosis as a critical feature of NAFLD is correlated with disease severity [12,13]. Moreover, diets with a high intake of fat, especially saturated fatty acids, promotes the development of NASH [14,15]. Palmitate (PA) as a saturated fatty acid could induce intracellular steatosis and cellular damage [13], which would be a risk factor for NAFLD. However, NAFLD presents different developmental stages and degrees of severity. The different degrees of injury in NAFLD indicate that there might be some protective factors against the injury.

Nearly a decade, research in autophagy has become overwhelming. Autophagy is discovered as an evolutionarily conserved to have vast array of homeostatic, developmental, and other physiological functions [16,17]. Autophagy, a cellular self-catabolic process, maintains cellular homeostasis by trafficking accumulation of damaged proteins and organelles to lysosomes for proteolytic degradation [18]. The interesting role of “self-eating” means it can break down harmful components from itself, thus showing a survival benefit. Moreover, it is regarded as a self-protective mechanism, coping with the cellular stress. Increasing evidence suggests that autophagy is involved in a broad spectrum of diseases. The study of Dutta D shows that autophagy induction can resist oxidative stress-mediated damage in cardiomyocytes [19]. Another research reported that human mesenchymal stem cells protected against apoptosis by enhancing autophagy in lung carcinoma cells [20]. Besides, autophagy activation can reduce renal tubular injury induced by urinary proteins [21]. According to the results from above studies, autophagy is taken as a benefit role in most situations. However, some researches also show that autophagy can promote cell death and the creation of apoptosis body [22]. Therefore, it is important to make it clear to the effect of autophagy in various situations. In the present research, we attempted to investigate the effect of PA treatment in hepatocytes and the role of autophagy in this process.

## Results

### PA induces hepatocytes apoptosis

**Figure 1:**
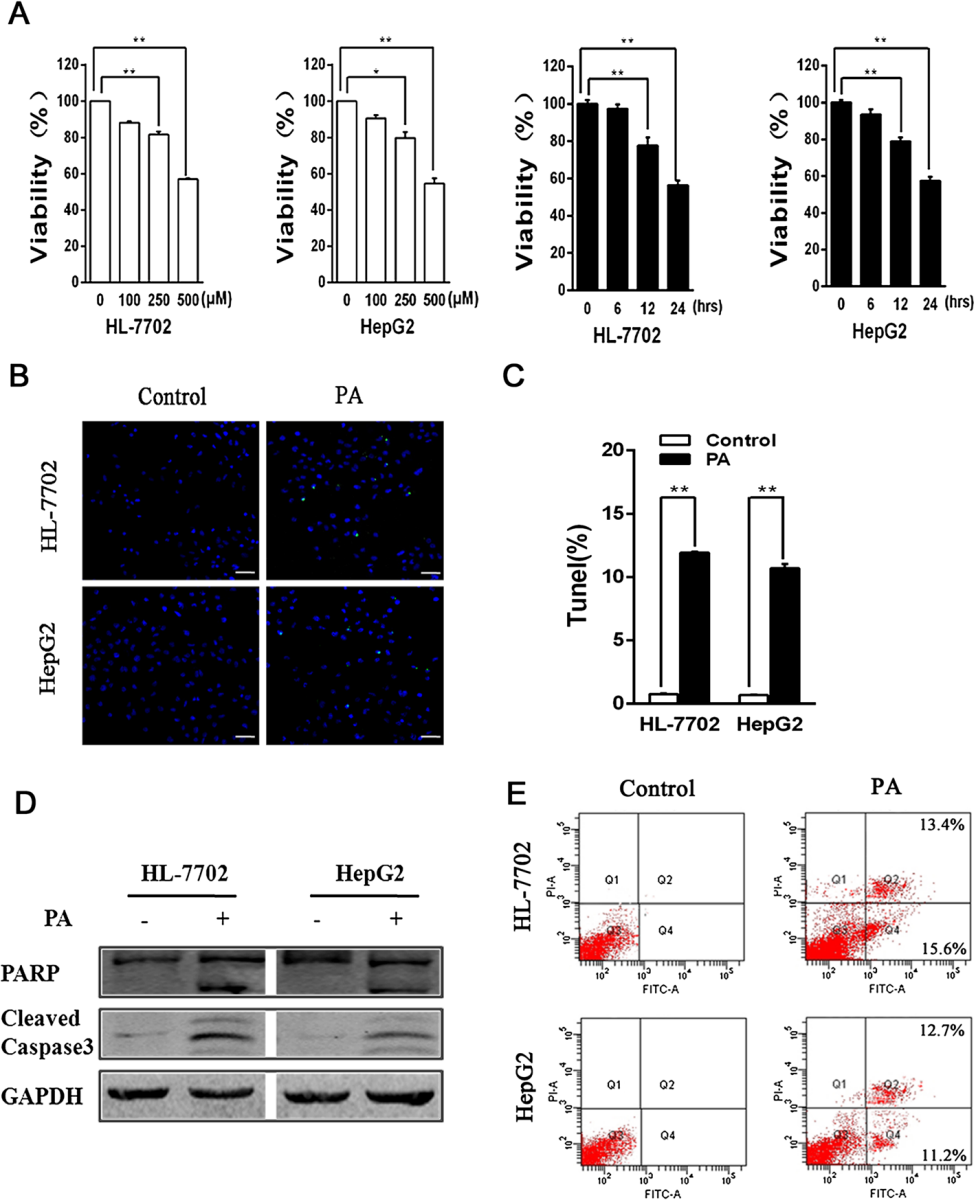
**PA stimulates apoptosis in hepatocytes. (A)** HL-7702 and HepG2 cells were treated with either control or PA (100 μM, 250 μM, 500 μM) for 24 hours. Cell viability was detected by CCK-8 assay. Respectively, at 0, 6, 12, 24 time points, PA (500 μM) and CCK-8 assay were used (*p < 0.05; **p < 0.01). **(B)** DNA fragmentation detection kit was used to deal with cells after treatment with control or PA for 24h, then cells were observed under a confocal microscopy (bar: 50 μm). **(C)** The ratio was calculated by counting the percentage of cells exhibiting positive TUNEL staining. Quantization was measured for the three times from the three times independent TUNEL assay (*p < 0.05; **p < 0.01). **(D)** Western blot analysis detected PARP and Cleaved-caspase3 proteins levels in cells after treatment with control or PA for 24 hours. **(E)** Cells were dealt with control or PA for 24 hours, and stained with AnnexinV-FITC and PI, and then apoptotic cells were quantified by flow cytometry (FCM). Numbers within quadrants represent the percentages of cells in early apoptosis ( AnnexinV + PI - ; lower right ) and in late apoptosis and necrosis (AnnexinV + PI + ; upper right ).

Various studies have shown that PA could cause cellular damage in some conditions. Here we tested whether a similar result occurred in hepatocytes with PA treatment. At first, we conducted the measurement of cell viability in HL-7702 and HepG2 cell lines. The result displayed a concentration dependency with PA treatment, and PA (250 μM or 500 μM) caused a marked reduction of cell viability. PA (500 μM) treatment also resulted in a gradual reduction of cell viability along with the increase of treatment time (Figure 1A). Moreover, treatment of PA brought about a marked increase in apoptotic cells (TUNEL-positive dots) in hepatocytes (Figure 1B and C). In further study, we performed western blotting analysis to evaluate the protein levels of two important apoptosis-associated factors, PARP and cleaved caspase3, in hepatocytes. As shown in Figure 1D, both cleaved PARP and cleaved caspase3 levels were markedly higher in PA treatment groups than in control treatment groups. In addition, Annexin V-FITC/PI staining analysis also demonstrated that PA treatment resulted in a significant increase of apoptosis in hepatocytes (Figure 1E). Taken together, these data suggest that PA induces apoptosis of hepatocytes.

### PA induces autophagy activation in hepatocytes

**Figure 2:**
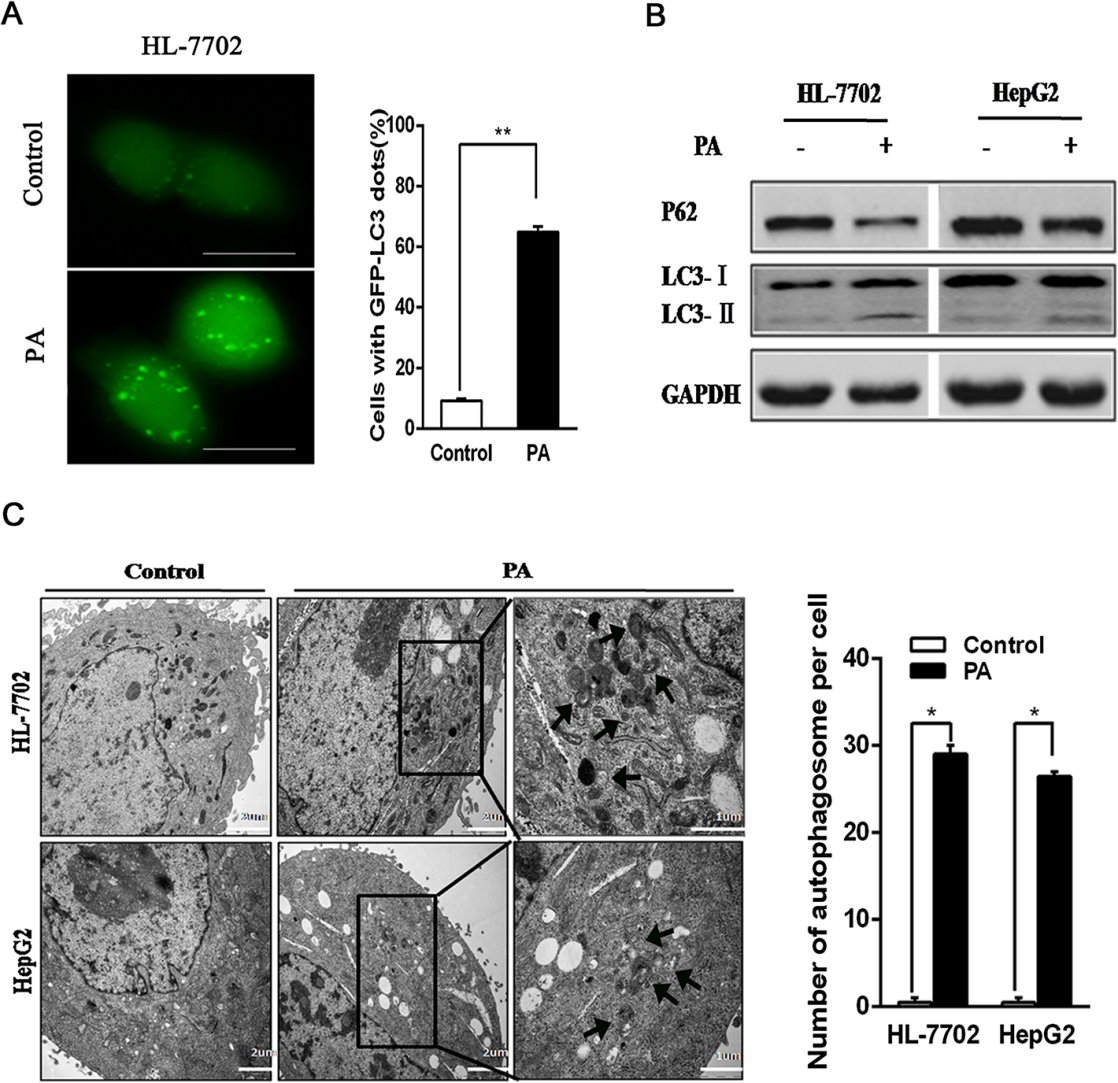
**PA stimulates autophagy activation in hepatocytes. (A)** HL-7702 cells were treated with control or PA for 24 hours, and plasmids of GFP-LC3 were transfected into the cells. Cells were observed under fluorescence microscope (bar: 20μm). Quantization was obtained by calculating the ratio of cells with GFP-LC3 dots in one visual field and experiments were repeated three times (*p < 0.05; **p < 0.01). **(B)** LC3 and P62 protein levels were detected by western blotting analysis after treatment of control or PA for 8 hours. **(C)** Cells were treated with control or PA for 8 hours before being processed, then electron microscope was performed at 11,500× and 29,500× magnification. The black arrows show membrane-bound vacuoles characteristic of autophagosomes. The number of autophagosomes per cell was quantitated. Date were presented as the mean ± SEM of three independent experiments (*p < 0.05; **p < 0.01).

Recent studies have reported that autophagy activation induced by PA occurred in MEF cells [17], INS-1E β-cells and isolated rat and human pancreatic islets [23]. To detect whether autophagy was activated by PA treatment on hepatocytes, we used GFP labeled microtubule-associated protein-1 light chain 3 (LC3) plasmid transfection in hepatocytes. PA stimulation resulted in an increase expression of autophagosomes (GFP-LC3 dots) in HL-7702 cell line under fluorescence microscope (Figure 2A). Meanwhile, we measured LC3 and p62 protein levels in hepatocytes. After eight hours of PA treatment, there was actually a remarkable increase of LC3II protein levels while P62 protein was degraded (Figure 2B). This might indicate that PA induced autophagic flux. To investigate autophagic levels, electron microscopy analysis was carried out in PA-treated hepatocytes. As shown in Figure 2C, many autophagosome structures were observed in hepatocytes by PA treatment. Nevertheless, we have not found the similar structure in control treatment groups. Therefore, these results indicate that PA also induces autophagy activation in hepatocytes.

### Autophagy inhibition augments apoptosis of PA-induced in hepatocytes

**Figure 3:**
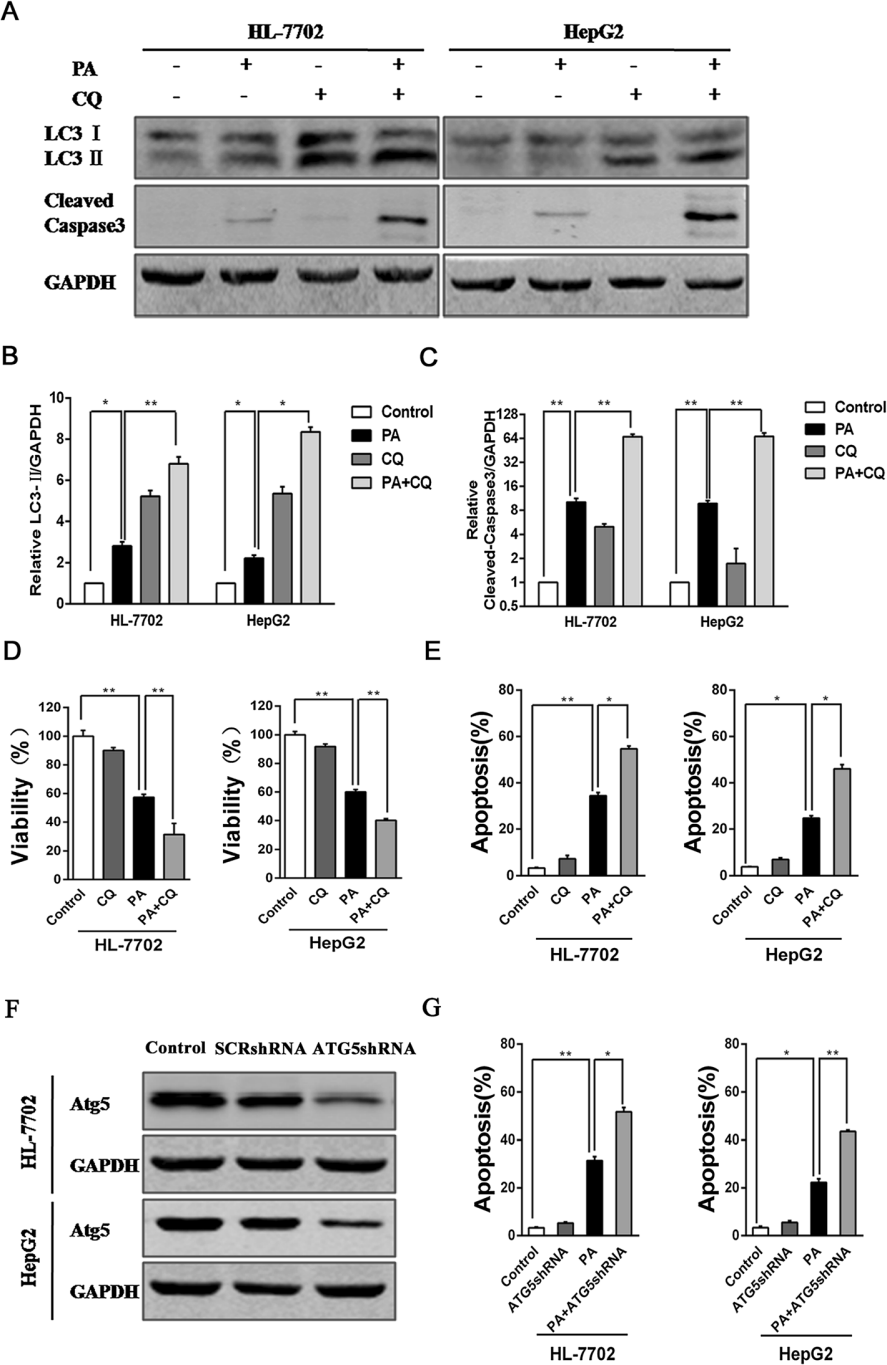
**Decreased autophagy level augments apoptosis of PA-induced in hepatocytes. (A)** Cells were treated with either control or PA for 24h. CQ (10 μM) was added to pretreatment for 8 hours. Western blotting was used for detecting LC3 and cleaved-caspase3 levels. **(B)** The relative LC3-II/GAPDH was calculated by normalizing their respective levels to the control level in cells. **(C)** The relative cleaved-caspase3/GAPDH was quantified in the same way. Date were presented as the mean ± SEM of three independent experiments (*p < 0.05; **p < 0.01). **(D)** Cells were quantified of the viability using CCK-8 assay after treatment with control or PA for 24h. CQ (10 μM) was also added to pretreatment for 8 hours. Data were repeated in three independent experiments and as the mean ± SEM (*p < 0.05; **p < 0.01). **(E)** Cells were treated in the same way, and then apoptotic cells were quantified by FCM after staining with AnnexinV-FITC and PI. The data represent the mean ± SEM values from three times separately (*p < 0.05; **p < 0.01). **(F)** Atg5 was knocked down with Atg5 shRNA infection in HL-7702 and HepG2 cells, then western blotting analysis were performed. **(G)** After cultured with control or PA for 24h, normal cells and the transfected cells were used to perform the apoptosis analysis by FCM. The data were expressed as the mean ± SEM values for three independent experiments (*p < 0.05; **p < 0.01).

Then we used chloroquine (CQ), a classical inhibitor of autophagy, to explore the role of autophagy in the PA-induced hepatocytes apoptosis. CQ can disrupt the fusion of autophagosome with lysosome and raise lysosomal pH to suppress the activity of lysosomal acid hydrolases, thereby blocking the degradation of autolysosome and accumulating LC3 II. CQ pretreatment resulted in accumulation of LC3 II in PA-treated or non-PA-treated hepatocytes (Figure 3A and B). Meanwhile, cleaved caspase3 expression levels were higher in combination of CQ pretreatment and PA treatment groups compared to those in PA treatment groups (Figure 3A and C). Pretreatment of CQ also led to decrease of cell viability in PA-treated hepatocytes (Figure 3D). In addition, FCM analysis revealed that CQ pretreatment brought about a significant increase in PA-induced cell apoptosis (Figure 3E). These data demonstrated that autophagy inhibition by CQ promoted PA-induced apoptosis in hepatocytes. In further study, we performed lentivirus-delivered shRNA to silence Atg5 expression in hepatocytes for disturbing autophagy. The data displayed that Atg5 expression levels were remarkmable lower in Atg5-shRNA transfected cells than in the non-transfected and scrambled shRNA (SCR-shRNA) transfected cells (Figure 3F). We also obtained the result the elevated apoptosis of PA-induced through FCM analysis in Atg5-shRNA transfected cells by inhibiting autophagy (Figure 3G). Taken together, autophagy inhibition augments PA-induced hepatocytes apoptosis.

### Autophagy activation reduces apoptosis of PA-induced in hepatocytes

**Figure 4:**
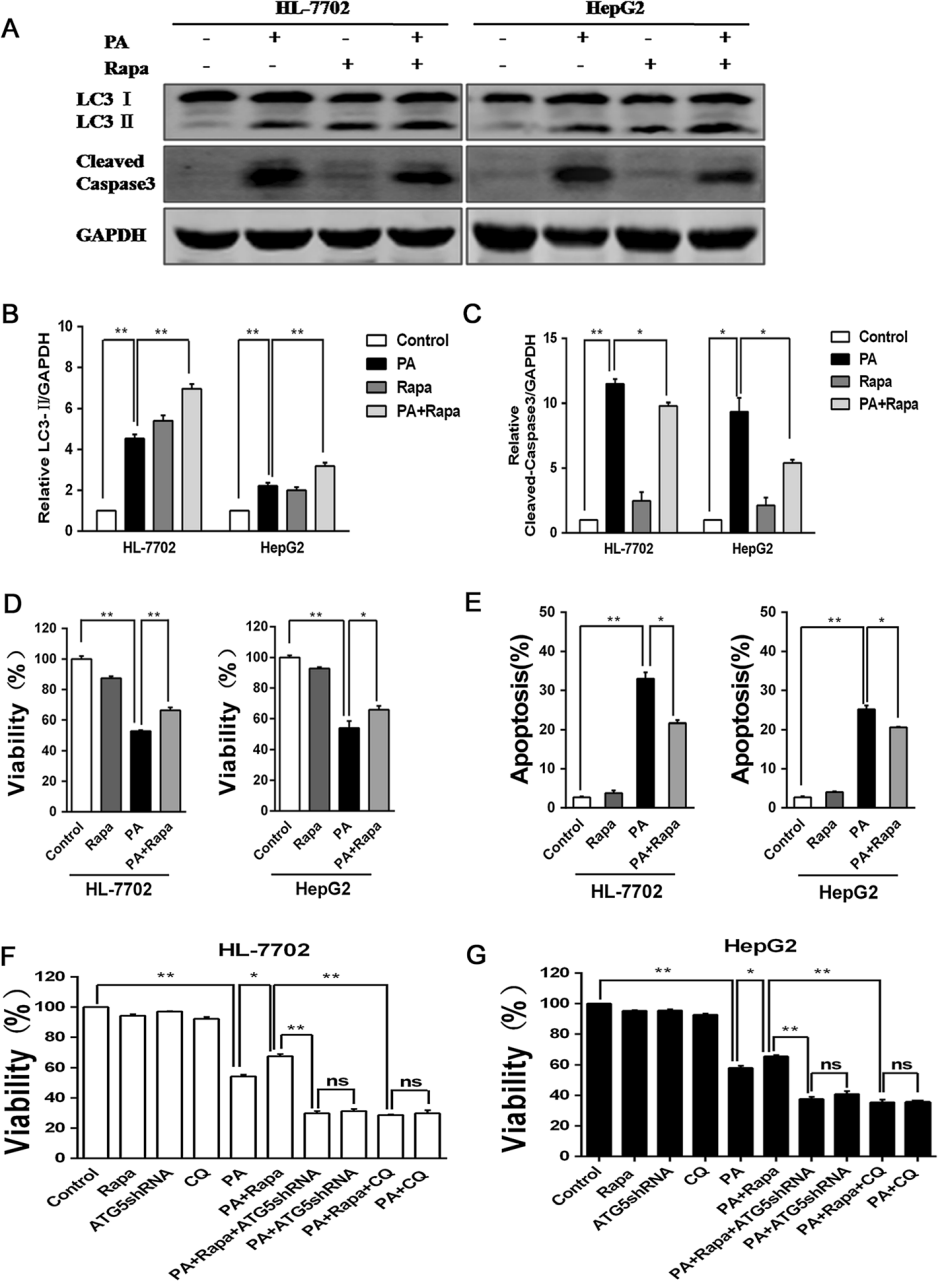
**Elevated autophagy level by rapamycin reduces apoptosis of PA-induced in hepatocytes. (A)** Cells were treated with either control or PA (500 μM) for 24h, Rapa (250 nM) was added to pretreatment for 8 hours, then western blotting analysis tested LC3 and cleaved caspase3 expressions levels. **(B)** The quantization of relative LC3-II/GAPDH from the treatment groups were calculated by normalizing to the control groups. **(C)** Relative cleaved-caspase3/GAPDH was calculated in the same way. Data were presented as mean ± SEM from three independent experiments (*p < 0.05; **p < 0.01). **(D)** Cells were treated with control, PA, Rapa (250 nM) and PA with the addition of Rapa pretreatment for 24 hours, then cell viability was detected by CCK-8 analysis. Data were mean ± SEM from the three times independent experiments (*p < 0.05; **p < 0.01). **(E)** Cells were stained with AnnexinV-FITC/PI after treatment as above, and were measured by FCM. The results were presented as mean ± SEM values at least three independent experiments (*p < 0.05; **p < 0.01). **(F)** HL-7702 cells were treated with various strategies, and then CCK-8 detected cell proliferation. **(G)** The result of cell proliferation in HepG2 cells was shown. Date were presented as the mean ± SEM values for three independent experiments (*p < 0.05; **p < 0.01).

We next explored the effect of activating autophagy in PA-induced hepatocytes apoptosis. Rapamycin (Rapa), a mammalian target of rapamycin (mTOR) inhibitor, has been used as a classical autophagy inducer. We found Rapa pretreatment enhanced the expression of LC3 II levels in PA-treated or non-PA-treated hepatocytes (Figure 4A and B). Meanwhile, cleaved caspase3 expression displayed a distinct lower levels in the combination of Rapa pretreatment and PA treatment groups than in PA treatment groups (Figure 4B and C). Besides, Rapa pretreatment effectively attenuated the decreased cell viability by PA treatment in hepatocytes (Figure 4D). The result from FCM analysis showed that Rapa pretreatment brought about the decrease of PA-induced apoptosis in hepatocytes (Figure 4E). These data demonstrated that autophagy activation by Rapa reduced cell apoptosis by PA treatment. In the further study, we performed cell viability assay with Rapa-/+ CQ and Rapa-/+ Atg5shRNA in hepatocytes. The result showed that Rapa treatment had no significant influence on the cell viability of CQ and PA combined treatment groups. Autophagy inhibition by Atg5-shRNA transfection revealed the similar result with CQ treatment (Figure 4F and G). These findings suggested that the effect of Rapa on promoting cell survival in PA-treated hepatocytes was due to autophagy activation. As a result, autophagy activation reduces PA-induced apoptosis in hepatocytes.

### PA induces PKCα activation, but has no influenced on mTOR and ER stress pathways in hepatocytes

**Figure 5:**
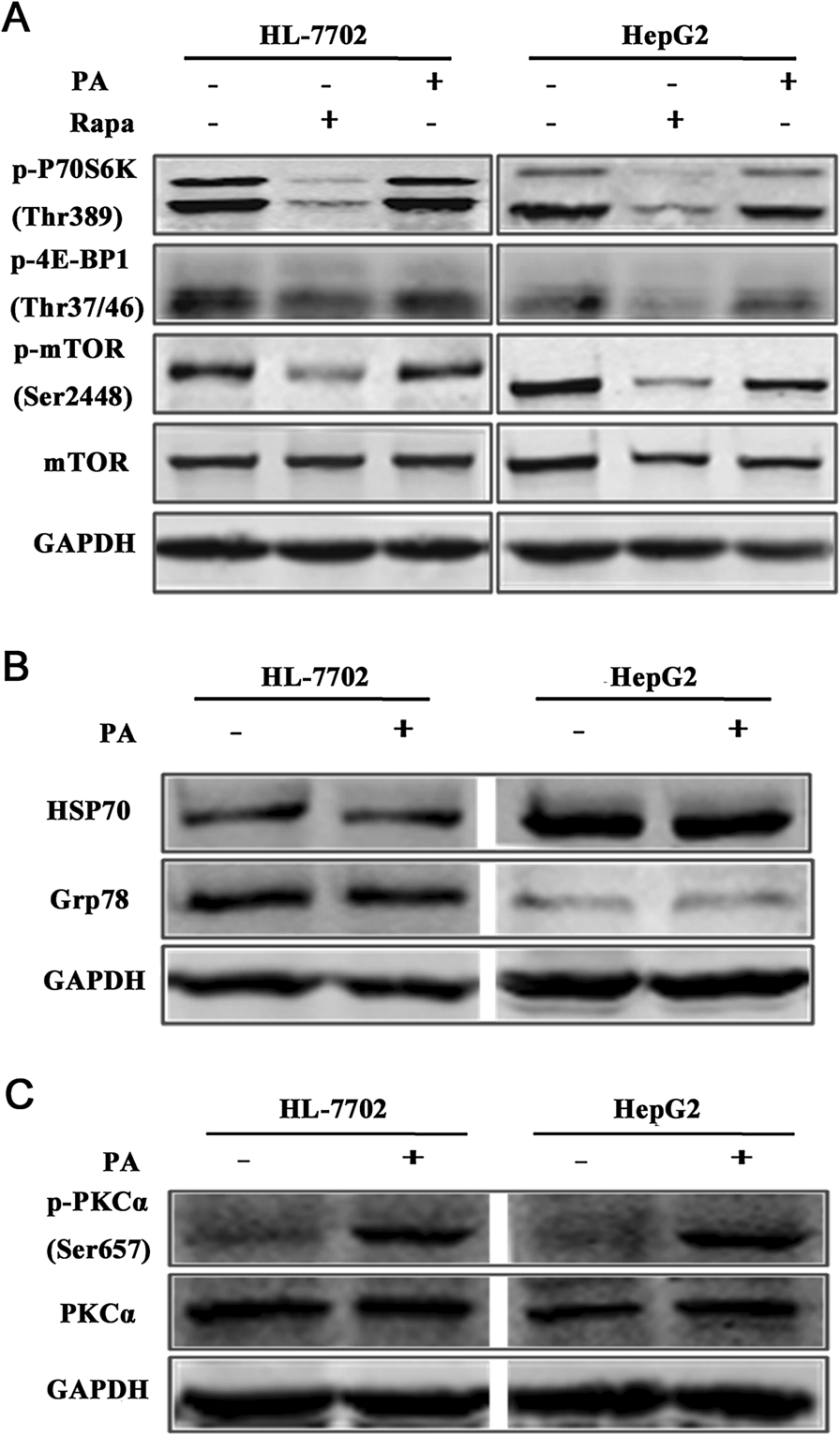
**PA induces PKCα activation, but has no influenced on mTOR and ER stress pathway in hepatocytes. (A)** The involvement of PA in mTOR signaling was analyzed by western blotting analysis after the treatment with either control or PA for 8 hours. Cells treated with Rapa (250 nM) for 8 hours were used as a positive control. **(B)** Western blot analysis was applied to detecting Grp78 and HSP70 proteins levels in cells after treated with Control or PA for 8 hours. **(C)** P-PKCα was measured by western blotting after with treatment as above.

In the further study, we explored the mechanism about PA-induced autophagy activation in hepatocytes. It was well-known that Rapa inhibited mTOR signaling pathway, thus activating autophagy. We wondered whether a similar pathway occurred in PA-induced autophagy activation, and then we primarily focused on mTOR signaling pathway. Western blotting analysis revealed that treatment of PA had no evident influence on p-mTOR levels, and meanwhile there was also no difference on phosphorylation of p70 S6 kinase (p70S6K) and 4E- binding protein 1 (4E-BP1) expression levels, as the two key downstream effectors of mTOR, in hepatocytes by PA treatment (Figure 5A). The study of Choi SE showed that endoplasmic reticulum (ER) stress could trigger PA-induced autophagy activation in INS-1 cells [24]. Therefore, we detected the expressions of two crucial ER stress markers HSP70 and Grp78 proteins, and the result suggested that PA did not cause significant difference on the two proteins levels (Figure 5B). In addition, ShiHao Tan et al. found that PA-induced autophagy activation was through protein kinase C (PKC)-mediated signaling pathway in MEF cells [17]. Our data also displayed that PA treatment led to elevated expression of p-PKCα levels, which demonstrated that PA activated PKCα in hepatocytes (Figure 5C). Therefore, PA might induce autophagy activation through activating PKCα pathway in hepatocytes.

## Discussion

In the present research, we found that PA could not only induce cell apoptosis but also activate autophagy in hepatocytes. Moreover, we also found that autophagy inhibition resulted in the elevated cell apoptosis of PA treatment, and in contrast activating autophagy brought about the decrease of PA-induced apoptosis in hepatocytes. In addition, it was also discovered that PA activated PKCα, and had no influence on mTOR and ER stress signaling pathways in hepatocytes. Together with these findings, we conclude that autophagy has an important role in protecting PA-induced hepatocytes apoptosis, and PA might activate autophagy through PKCα pathway in hepatocytes.

Laura L. Listenberger et al. reported that PA-induced apoptosis occurred in Chinese hamster ovary cells via the generation of reactive oxygen species [25]. Taheripak G and his colleagues found that PA could induce mitochondrial dysfunction and apoptosis in skeletal muscle cells [26]. In addition, some research reports that PA induces hepatocytes lipoapoptosis [27-29]. These reports are identified with the damage effect of PA. We also found that PA led to apoptosis in hepatocytes, and autophagy could be activated with PA treatment. Moreover, through the effect of regulating autophagy, we have proved that autophagy had a protective effect in PA-treated hepatocytes. Autophagy was reported that it has a pro-survival function under stressful “life-threatening” conditions in most liver disease [30]. Song MY et al. discovered that dimethyl sulfoxide reduced hepatocellular lipid accumulation by autophagy induction [31]. Consequently, autophagy played a protective role in PA-induced hepatocytes apoptosis.

The reason why PA was able to activate autophagy in hepatocytes was speculative. Blocking mTOR signaling is the best pathway for activating autophagy [32]. P70S6K and 4E-BP1 are two crucial downstream substrates of mTOR signaling. When sufficient nutrients are available, mTOR is phosphorylated and transmits a positive signal to p70S6K and the inactivation effect of 4E-BP1 [33]. We found that PA treatment caused no significant difference in phosphorylation levels of mTOR, p70S6K and 4E-BP1 in hepatocytes, in comparison to control treatment. Therefore, PA-induced autophagy activation in hepatocytes was independent of mTOR signaling pathway. Accumulating data indicated that ER stress was a potent trigger of autophagy [34-37], and FFAs have been reported to have a function of generating ER stress in hepatocytes [38]. Nevertheless, our result was not consistent with these findings, since PA had no influence on ER stress markers in hepatocytes, suggesting that autophagy activation was independent of ER stress pathway. Then PKCα, as a member of the classical PKC family, was found played a critical mediator in PA-induced autophagy in MEF cells [17]. We investigated the role of PKCα in hepatocytes with PA treatment. It was found that PA treatment activated p-PKCα in hepatocytes. Taken together, PA might activate PKCα pathway for activating autophagy in hepatocytes.

In conclusion, PA can induce hepatocytes apoptosis and during the process autophagic system is activated, and the activated autophagy plays a protective role against PA-induced apoptosis. Besides, PA might induce autophagy through activating PKC α pathway in hepatocytes. However, the detailed mechanism involved in the protective effect of autophagy in PA-treated hepatocytes has yet to be further research.

## Materials and methods

### Materials

PA, Albumin from bovine serum (BSA, fatty acid free) and CQ were purchased from Sigma-Aldrich (St.Louise, MO). Rapamycin was purchased from Gene Operation Datasheet. Cell counting kit-8 (CCK-8) assay kit was purchased from DOJINDO (Japan). AnnexinV/PI analysis kit was purchased from KeyGen Biotechnology (China). DAPI staining solution was purchased from Beyotime Institute of Biotechnology (China). GAPDH was purchased from HuaAn Biotechnology (China). RIPA buffer and other all antibodies were purchased from Cell Signaling Technology (Beverly, MA). Pierce BCA Protein Assay Kit was purchased from Thermo Fisher Scientific. Fugene HD transfection reagent was purchased from Roche (04709705001). Odyssey Blocking Buffer was purchased from LI-COR Biosciences. DNA fragmentation detection kit was purchased from Calbiochem (America).

### Cell culture

HL-7702 cell was maintained in RPMI 1640 medium supplemented with 10% fetal bovine serum, 100 U/ml penicillin and 100 g/ml streptomycin at 37°C in a humidified atmosphere containing 5% CO_2_. HepG2 cell was maintained in DMEM medium supplemented with the same conditions. Above reagents were purchased from Gibco Life Technologies.

### Preparation of PA

Briefly, 0.103 g Palmitic acid was prepared in 0.1 M 200 ml NaOH at 70°C and filtered. Five percent FFAs-free BSA solution was prepared in double-distilled H_2_O and filtered. The solution of PA was conjugated to 5% BSA in a 70°C water bath. The above solution was then cooled to room temperature and diluted in RPMI 1640/DMEM to final concentrations [39]. Cells were treated at the concentration of 500 μM PA in the present research normally. Cells were cultured in RPMI 1640/DMEM with 3% FBS as control.

### Cell viability assay

Cells (5 × 10^3^ cells/well) were seeded in 96-wells plate, and cultured overnight. After treatments as indicated, cells were incubated with the mixed liquor (10 μL CCK-8 reagent + 90 μL RPMI 1640/DMEM medium) at 37°C for 1 hour. Then the value was measured at 450 nm of light absorption.

### TUNEL assay

Cells were seeded in microscope slides, and then were placed in 24-wells plate. After treated as indicated, cells were fixed using 4% paraformaldehyde, and the manufacturer’s protocol was followed. TUNEL positive cells were observed under confocal microscopy.

### Western blot analysis

Cellular protein was extracted with 1× cell RIPA buffer. Density of proteins was determined by Pierce BCA Protein Assay Kit. According to the routine, equivalent amounts of protein (30 μg) were loaded onto poly-acrylamide gels, electrophoresed, and then transferred onto nitrocellulose NC membranes (Whatman). After blocking these membranes with odyssey blocking buffer for 1 hour, target antigens were reacted with primary antibodies and subsequently secondary antibodies. At last, the membranes were scanned by the Odyssey infrared imaging system.

### Transfection of GFP-LC3 plasmids

Cells were seeded in 96-well plates, then GFP-LC3 expression plasmids were transfected into the cells using Fugene HD transfection reagent. After 24 hour, cells were treated with PA (500 μM) or non-PA for 24 hours. Autofluorescence GFP-LC3 was observed under fluorescence microscope.

### Gene silencing with lentivirus-delivered shRNA

shRNA candidate target sequence to Atg5 is 5′-CCTTTCATTCAGAAGCTGTTT-3′. Scrambled shRNA sequence, as a negative control, is 5′-TTCTCCGAACGTGTCACGT-3′. The oligonucleotides encoding the Atg5-shRNA or Scrambled shRNA sequence were inserted into the GFP express vector pGCL-GFP (Shanghai GeneChem, shanghai, china). The recombinant virus was packaged using Lentivector Expression Systems (Shanghai GeneChem). HL-7702 and HepG2 cells were infected, and observed under fluorescence microscope after 72h.

### Annexin V-FITC and PI Staining Analysis

In order to assess apoptosis, 1× 10^6^ cells were plated onto 6-well culture plates and treated with ligands previously. Following staining according to manufacturer’s protocol, the apoptosis analysis of cell was performed by flow cytometry (FCM).

### Statistical analysis

All the data were expressed as mean ± SEM deviation of at least three independent experiments. Statistical differences between the various groups were compared by using Student’s t test and one-way ANONA. P values less than 0.05 were considered statistically significant.

## Abbreviations

NAFLD: Non-alcoholic fatty liver disease; NASH: Nonalcoholic steatohepatitis; FFAs: Free fatty acids; TAGs: Triacylglycerols; PA: Palmitate; FCM: Flow cytometer; CQ: Chloroquine; LC3: Microtubule-associated protein 1 light chain 3; Rapa: Rapamycin; mTOR: mammalian target of rapamycin; ER: Endoplasmic reticulum; p70S6K: p70 S6 kinase; 4E-BP1: 4E- binding protein 1; PKC: Protein kinase C; CCK-8: Cell counting kit-8.

## Competing interests

The authors declare that they have no competing interests.

## Authors’ contributions

NC and XZ contributed equally to this manuscript. NC, YYJ, KS and LXW designed the study. XZ and N C performed cell experiments. XZ and SFJ performed molecular experiments. XJC and YZ performed the apoptosis analysis by flow cytometry. HZY and NC performed the experiment of confocal microscope. NC and XZ wrote the draft manuscript. LXW finalized the manuscript. All authors read and approve the final manuscript. All authors read and approved the final manuscript.
